# Interculturality in the Development of Technology-Mediated Courses for Massive Health Education: A Systematic Review

**DOI:** 10.3390/ejihpe14100181

**Published:** 2024-10-08

**Authors:** Priscila Sanara da Cunha, Ingridy Marina Pierre Barbalho, Felipe Ricardo dos Santos Fernandes, Manoel Honorio Romão, Janaina Luana Rodrigues da Silva Valentim, Karla Mônica Dantas Coutinho, Kaline Sampaio de Araújo, Ricardo Alexsandro de Medeiros Valentim, Aline de Pinho Dias, Natalia Araújo do Nascimento Batista, José Adailton da Silva, Heleni Aires Clemente, Maria de Fátima Pereira Alves, Karilany Dantas Coutinho

**Affiliations:** 1 Laboratory for Technological Innovation in Health (LAIS), Federal University of Rio Grande do Norte, Natal 59012-300, RN, Brazil; ingridy.marina@lais.huol.ufrn.br (I.M.P.B.); felipe.ricardo@lais.huol.ufrn.br (F.R.d.S.F.); manoel.romao@lais.huol.ufrn.br (M.H.R.); janaina.lrsv@lais.huol.ufrn.br (J.L.R.d.S.V.); karla.coutinho@lais.huol.ufrn.br (K.M.D.C.); kaline.sampaio@lais.huol.ufrn.br (K.S.d.A.); ricardo.valentim@lais.huol.ufrn.br (R.A.d.M.V.); aline.dias@lais.huol.ufrn.br (A.d.P.D.); adailton@lais.huol.ufrn.br (J.A.d.S.); heleni.aires@lais.huol.ufrn.br (H.A.C.); karilany@lais.huol.ufrn.br (K.D.C.); 2 Intercultural Relations Graduate Program, Open University of Portugal, 1269-001 Lisbon, Portugal; fatimaa@uab.pt; 3 Centre for Interdisciplinary Studies—CEIS20, Faculty of Arts and Humanities, University of Coimbra, 3000-186 Coimbra, Portugal; 4 Department of Biomedical Engineering, Federal University of Rio Grande do Norte, Natal 59078-900, RN, Brazil; 5 Health Management and Innovation Graduate Program, Federal University of Rio Grande do Norte, Natal 59078-900, RN, Brazil; 6 Department of Educational Fundamentals and Policies, Federal University of Rio Grande do Norte, Natal 59078-900, RN, Brazil; 7 Department of Collective Health, Federal University of Rio Grande do Norte, Natal 59078-900, RN, Brazil; 8 Faculty of Health Sciences of Trairi, Federal University of Rio Grande do Norte, Santa Cruz 59200-000, RN, Brazil; 9 Centre for Functional Ecology—Science for People & the Planet, TERRA Laboratory, Department of Life Sciences, Faculty of Sciences and Technology, University of Coimbra, 3000-456 Coimbra, Portugal

**Keywords:** virtual learning environment, MOOC development, interculturality, health training, distance education, technology-mediated education

## Abstract

Virtual Learning Environments have become innovative tools in health professionals education. Through Massive Open Online Courses, they enable different ways of connecting with knowledge, facilitating study autonomy, interaction, and closer alignment with professional practices and the context of course participants. MOOCs comprise an educational strategy for many fields, including health. As they educate health professionals about a variety of practices, MOOCs play a crucial role in interculturality by enabling professionals to approach cultural diversity in work settings. This study provides a review of the literature investigating the element of interculturality in the production of healthcare-related MOOCs intended for a variety of audiences, including practicing healthcare professionals, healthcare professional trainees, and the general public. Based on a systematic review protocol, we searched for peer-reviewed studies published between 2016 and 2023 in Science Direct, PubMed, ERIC, and Scopus. Fifteen studies were selected for final analysis, which highlighted MOOC development, its underlying processes, and its importance in promoting health and social well-being. MOOCs have (1) provided new approaches to technology-mediated learning in distance health education, (2) aided training, (3) disseminated knowledge, and (4) promoted interculturality. Continuous collaboration and innovation in MOOC development are essential to ensure their effectiveness and relevance in the contemporary educational scenario.

## 1. Introduction

Health practices and care are significantly influenced by the cultural (including customs, beliefs, and religions), historical, social, political, and economic aspects of the territory in which the health professional works. In this process, interculturality appears as a fundamental element to be considered in health professional education. The implementation of cultural safety and anti-racist practices, as well as the promotion of equity in medical care, are actions that can reduce health disparities. In addition, they can improve communication between healthcare professionals and patients from different cultural backgrounds to create a more inclusive and equitable healthcare environment [1].

The concept of interculturality was coined to represent a set of ideas aimed at democratic coexistence between diverse cultures. Martín et al. [2] describe it as the process of interaction and exchange between different cultures aimed at mutual understanding, valuing diversity, and building peaceful and harmonious coexistence. From this perspective, interculturality involves recognizing and respecting cultural differences and promoting intercultural dialog and cooperation between diverse groups.

In Raymundo’s view [3], promoting interculturality means pursuing contextualized dialog based on the experiences of each interlocutor to achieve effective and not just superficial communication. Some factors contribute to interculturality in its broadest sense, e.g., knowing the other person’s culture, recognizing one’s own culture, eliminating or neutralizing prejudices, establishing empathetic relationships, and recognizing metacommunication, which is not an obvious element. Put succinctly, metacommunication involves the investigation and analysis of implicit and non-verbal messages, which are often subtle and not obvious [4].

Interculturality encourages the construction of an inclusive and plural society in which diverse cultural identities are recognized and valued. However, the realization of such a society is confronted with significant challenges. They relate to the existence of stereotypes, prejudices, inequalities, contradictions, and conflicts. There are also those factors that hinder metacommunication, such as ignorance, universalization based on one’s concepts, and the overvaluation of differences.

In line with the perspectives of cultural diversity and interculturality, the United Nations Educational, Scientific and Cultural Organization (UNESCO) has been working towards recognizing equality and the definition of intercultural diversity to adopt new social and humane policies [5]. Interculturality aims not to separate the universal from the particular but to bring together and organize an integrative social and relational context. Therefore, it is necessary to consider this pluralism to promote dialog between different types of knowledge since the common objective between them is to seek balance or health through care [3].

The intercultural approach to health, or interculturality in health, means the set of actions and policies that seek to recognize and incorporate the user’s culture into the healthcare process. The cultural relevance of such a process surpasses the exclusively ethnic dimension, as it implies valuing biological, cultural, and social diversity as essential elements in all health and disease processes [2]. This is attributable to the strong influence of cultural, historical, social, political, religious, and economic aspects on health care [3,6]. Therefore, the intercultural approach comprises an innovative way of considering the aspects that impact individual well-being.

When it comes to health professional education, interculturality elements are relevant for the promotion of learning environments with enriching interactions between professionals from different cultural backgrounds [7]. This could help train health professionals that provide more empathetic and effective care, respecting the uniqueness of each individual’s cultural context.

Owing to the importance of interculturality in promoting equity in health and in training professionals capable of recognizing and valuing cultural diversity, there is a need to investigate the presence and effectiveness of intercultural elements in courses offered by Virtual Learning Environments (VLEs), looking at the production process of MOOCs in health. In turn, intercultural dialog is relevant not only among distinct cultures and identities but also across different knowledge fields. Thus, interculturality in health can be seen as the complementarity between various perspectives on the same health problem, established through dialog and exchange. It is about the coexistence of different worldviews in a complementary way, without prejudice or the imposition of one over the other [3].

Health training is increasingly recognized as a fundamental element in improving the clinical practices of health professionals to promote individual and collective health in Indigenous and, above all, intercultural contexts for the definition of health policies [8]. Over the last two decades, technology-mediated training has been adopted as an essential strategy for promoting health education [9]. VLEs are a case in point, as they have been used to promote innovative educational practices in the training of health professionals and the general population. This allows new ways of connecting with knowledge, autonomy in study and interaction, as well as greater proximity to professional practices and specific contexts.

VLEs provide the environment for the dissemination of Open Educational Resources (OERs). OERs are “learning, teaching and research materials in any format and medium that reside in the public domain or are under the copyright that has been released under an open license, that permits no-cost access, re-use, re-purpose, adaptation and redistribution by others” [10]. OERs on health topics can be developed to meet the specific needs of different population groups, offering culturally sensitive information adapted to different socioeconomic contexts. Some examples include OERs that cover the prevention, diagnosis, and treatment of syphilis and other Sexually Transmitted Infections (STIs) or learning pathways aimed at the prison system [11]—with OERs adapted for very specific audiences such as people deprived of liberty, prison officers, and health workers operating in prisons.

Courses known as Massive Open Online Courses (MOOCs) are hosted in VLEs with the intent to broadly disseminate information, including OERs. They are scalable, offer learners the flexibility of time and space, and can disseminate information rapidly to address public health crises and facilitate intercultural dialog. MOOCs are designed to involve many participants in remote learning, which can include health care related topics. MOOCs are also a means for social inclusion, as they are efficient for massively disseminating educational content across diverse groups globally [12].

The adoption of educational practices supported by MOOCs has openly boosted the dissemination of knowledge free of charge in certain VLEs, contributing to the promotion of a culture of sharing and collaboration. In the field of health, this dissemination of knowledge is especially relevant, as it allows important information on prevention, treatment, and health care to reach a wider and more diverse audience [8]. In this sense, access to OERs has significantly contributed to tackling health crises and emergencies worldwide, reaching different regions and cultures in a broad, accessible, and equal way [13].

In this context, it is essential to develop expertise in the production of educational resources that meet the demands of training in the health area. Education mediated by technology has peculiarities that require greater care regarding communication and the use of Information and Communication Technologies (ICTs) in the teaching-learning process, in pedagogical strategies, and in the educational methodologies necessary for the construction of knowledge [11]. Thus, creating a MOOC requires a careful construction of teaching material.

MOOC production in the health field entails several stages, including planning, content development, technical review, instructional design, and platform integration. In the context of continuing health education, the process aims to improve distance learning for health professionals, ultimately enhancing their ongoing training and professional practices [14].

In view of the above, this study aims to conduct a systematic literature review to investigate the element of interculturality in the production of MOOCs in the health field, exploring the role of these courses as fundamental tools for promoting the education process of health professionals.

## 2. Materials and Methods

This review followed the systematic review guidelines proposed by Kitchenham [15] and the Preferred Reporting Items for Systematic Reviews and Meta-Analyses (PRISMA) checklist [16]. Systematic Literature Reviews (SLRs) provide a structured analysis of the available evidence and assist in detecting patterns, gaps, and trends within the existing body of literature. In addition, they permit comparisons of practices, methodologies, and results across different studies, providing valuable insights into the research practices undertaken and indicating new directions for future research [17].

Our primary focus was to investigate the element of interculturality in the production of MOOCs in the health field, emphasizing the role of these courses as fundamental tools for promoting the education process of health professionals. We also sought to identify features of the development process, such as production planning, content definition, target audience, platform for provision, platform for access, and definition of the professionals involved in MOOC development [11,18]. Four research questions (RQs) were defined to obtain relevant information and achieve the research objective (see Table 1).

Articles included in this review were retrieved from the ScienceDirect, PubMed, ERIC, and Scopus databases in March 2023. The following search string was applied: (“distance education” or “virtual learning environment” or “VLE”) AND (“massive open online courses” or “MOOC”) AND (“human training in health” or “health education”). The selection process was performed in three stages: (i) identifying and organizing the articles; (ii) screening the articles according to inclusion and exclusion criteria; and (iii) analyzing the articles according to their quality.

In stage (i), the first set of articles returned from the database search was identified. In stage (ii), inclusion and exclusion criteria were defined (see Table 2). The set of articles obtained in stage (i) was screened according to the inclusion criteria (IC) applied in the databases. Subsequently, exclusion criteria (EC) were applied with the aid of the Rayyan web platform [19], generating the set of articles for quality criteria assessment.

We included peer-reviewed articles published between 2016 and 2023. The decision to include studies published from 2016 onwards follows the Qingdao Declaration from UNESCO [20]. The declaration calls for the development of comprehensive strategies and training programs to harness the potential of OERs and promote the integration of ICT into education. The IC01 was also based on the need to capture and analyze the current state of practice and research in the field under investigation. The timeframe analyzed enabled a comprehensive view of emerging trends, technological innovations, and changes in educational approaches considering cultural diversity [21]. By concentrating on recent studies, the review sought to update knowledge of how different cultures are integrated into VLEs, identifying successes, challenges, and shortcomings. In addition, such temporal delimitation facilitated comparison across studies, providing consistency to the criteria and valuable insights for future educational practices and research.

During stage (iii), the articles retrieved from the previous stages were fully screened and assessed according to the quality assessment (QA) criteria (see Table 3). At this stage, the articles were assessed using the arithmetic mean of the weights (w) assigned to each quality assessment criterion, according to Equation (1) [13].

(1) $$score=\frac{1}{n_{QA}}\;\sum_{i=1}^{n_{QA}}\;w_{{QA}_{i}}$$

where:

- *n_QA_*: total number of criteria for QA;
- *w_QA_*: value corresponding to the weight assigned to the QA criteria under analysis.

**Table 3:** Quality Assessment Criteria.

No.	Description
QA01	Does the study address the production process and stages of MOOCs in the field of health?
QA02	Does the study address the main elements/characteristics applied to interculturality in MOOC development?
QA03	Does the study address the contribution of MOOCs to improving Distance Education actions in the field of health?
QA04	Does the study address the methodologies adopted in MOOC production? Was interculturality incorporated into such methodologies?

The weight value measures the extent to which a study meets a given QA criterion, with 1.0 being the most relevant weight and 0 the least relevant, according to Equation (2). Primary articles scoring 0.5 or higher were considered eligible (i.e., 0.5 ≤ *score* ≤ 1.0).

*wQA* = 1.0, yes, it fully describes,
0.5, yes, it partially describes,
0, it does not describe.(2)

**Figure 1:**
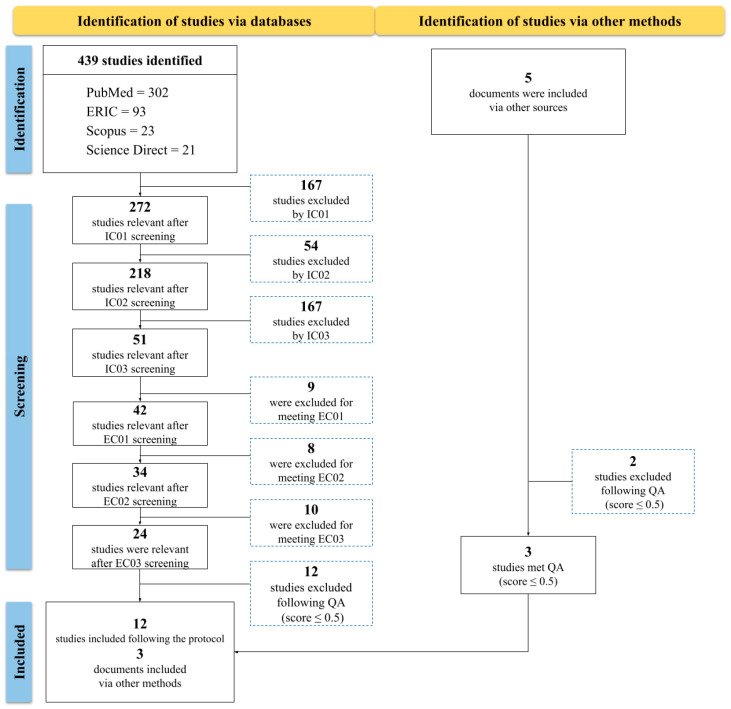
Protocol for identifying, screening, and including studies in the review.

Studies from other sources were included via another method, where a further search included studies on health education. To accomplish this, search methods were applied in academic research platforms, including SciELO, Educapes, and EDUFRN. These searches were actively conducted, employing the keywords “health education” and “MOOC”. This process made it possible to widen the research scope, as well as to incorporate relevant studies, therefore contributing to a more comprehensive approach to the subject. Figure 1 describes how the study search and selection process was conducted.

## 3. Results

In the identification phase, the search conducted in the established databases resulted in 439 selected articles (Figure 1). Subsequently, IC and EC were applied, as well as the QA criteria used for study selection. This resulted in a sample of 24 articles for analysis. From this sample, 12 were excluded during evaluation for not reaching the minimum score stipulated (score ≤ 0.5). Finally, 12 articles remained for inclusion in the review.

Moreover, five additional articles were identified through an independent search strategy to broaden the research scope and include other relevant studies in health education. These articles underwent the QA process, resulting in the exclusion of two studies due to insufficient scores. This resulted in three being selected for the review. At the end of the process, 15 articles were selected for a detailed analysis with the aim of answering the research questions.

**Figure 2:**
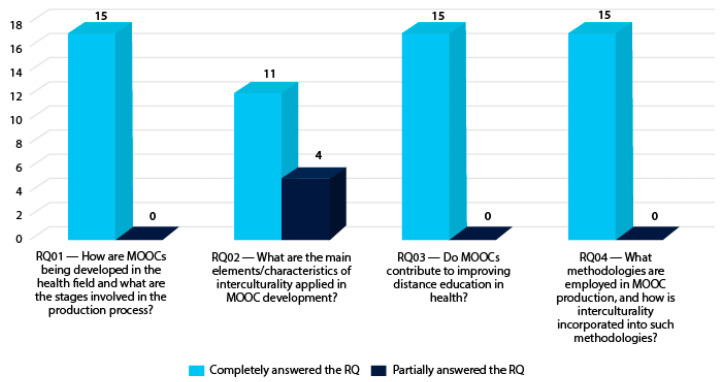
Analysis of the studies in the systematic review according to the RQ.

Figure 2 shows an analysis of the references included in the systematic review in terms of the RQ. As mentioned, a total of 15 articles were selected that addressed the issue of how MOOCs are being produced; 11 of them discussed elements and characteristics of interculturality applied in the MOOC production process; 15 were found to have contributed to a process of technology-mediated education in the health field; and 15 indicated the methodologies used in the MOOC production process and how the theme of interculturality is reflected in these methodologies.

Table 4 shows the 15 studies selected for this review, describing the title, authors, and year of publication. The journals were peer-reviewed and published in English and/or Portuguese. The studies covered the production process of MOOCs, aspects of interculturality, and the benefits of distance health education, presenting information relevant to achieving this research goal. In this context, the research questions are answered below.

### 3.1. RQ01—How Are MOOCs Being Developed in the Health Field, and What Are the Stages Involved in the Production Process?

According to the 15 studies included in this systematic review, the production of MOOCs in the health field has been undertaken by teams, professors, and professionals within the field in a series of stages. These stages range from planning to launching the course on an online platform.

On the platform of the Virtual Learning Environment of the Brazilian Health System (AVASUS), developed by the Laboratory for Technological Innovation in Health (LAIS) of the Federal University of Rio Grande do Norte (UFRN), the production of MOOCs follows certain criteria for the development of courses.

MOOC development includes a series of important stages, such as course planning, training of content creators, content development, technical and scientific review, pedagogical review, instructional design—i.e., how the content will be presented to course participants—, language style standardization, educational resources formatting, integration into the VLE, validation of the content to ensure its quality, and the final launch. All these stages involve teams from different fields, such as education, communication, design, health, information technology, engineering, accessibility, and linguistics [23].

For instance, this process includes the ADDIE model, a methodology that follows five stages: Analysis, Design, Development, Implementation, and Evaluation, which has been applied to the Moodle platform. Notably, renowned institutions such as the Open University of Portugal, the University of London, the University of Puerto Rico, New York University, the University of Edinburgh, and the University of Queensland have adopted this model.

According to Rodrigues & Rossi [32], the ADDIE production model is characterized by the analysis of course objectives, verification of the target audience, and definition of skills and knowledge. The aim is for participants to be guided and to set realistic expectations of the course, contributing to a more efficient and satisfying learning experience, and for the course design to ensure that the content is well-structured, engaging, and effective in sharing the intended knowledge [32].

From the perspective of MOOC production in the health area, i.e., the focus of this study, Valentim et al. [34,35] highlighted the need to increase scalability. It pertains to the reach of these courses when offered on the platforms, as they must reach many health professionals across the different geographical regions of the country. In addition, the authors pointed out the need to develop strategies in the production process, such as more dialogical and enjoyable content to fit in with the study routine of these professionals and reflect the dynamic reality of the Brazilian Health System (SUS). It is worth noting that the dialogical approach involves the creation of content that promotes interaction and exchange of ideas among MOOC students, facilitating active and participatory learning.

Longhini et al. [27] mapped data through a sample, making it possible to identify trends, patterns, and common characteristics of MOOCs. This study used methods and tools to measure learning outcomes and factors that increased the effectiveness of the courses, ensuring continuity of education in undergraduate and postgraduate programs in the Health Sciences. The students’ motivation and involvement led to a high level of knowledge, skills, and health literacy.

Some authors show that the courses produced went through stages of translation before being published on the VLE platforms [11,25,26,28,30,31]. In other words, there was a need for another stage in the production process to make the content more accessible when it comes to issues related to language and local culture.

According to the articles analyzed in this review, MOOCs have been developed following sequential production stages by means of prior planning according to local and cultural needs, whether these are those of the region or the country. After the planning stage, the content was developed in its raw format. The educational resources were then developed using elements such as videos, texts, clinical trials, design, accessibility, and other formats. It is important to note that the accessibility features included audio volume control for people who are hard of hearing, the use of voice commands on the computer, and biometric alternatives, which are technological combinations that facilitate communication, the learning process, and social inclusion. The final stage of this process consists of evaluation, followed by making the course available to the public on the VLE.

### 3.2. RQ02—What Are the Main Elements/Characteristics of Interculturality Applied in MOOC Development?

By analyzing the selected articles, it was possible to identify some characteristics and elements applied in the production process of MOOCs involving the theme of interculturality. In the studies, interculturality was taken into account and used during the content proposal according to the need to produce and offer courses. The purpose was to reach a specific audience to promote policies and good practices, interaction, and understanding on the subject, as well as respect between different cultures and groups.

In these studies, accessibility, global reach, diversity of participants, flexibility, autonomy, technology, and interactivity were all strengths of MOOCS. In terms of limitations, we observed superficiality in interactions, inequalities in access to technology, challenges in moderation and mediation, and cultural context limitations. These were related to ineffective communication, which could not encompass cultural differences, thus hindering mutual understanding and homogeneity of teaching materials.

The MOOCs reported by Caitano et al. [11] included people in situations of vulnerability, including LGBT people (Lesbian, Gay, Bisexual, Transgender), men who have sex with men (MSM), and people deprived of liberty. In addition, the MOOCs also contain topics related to syphilis in pregnancy and prenatal care, vertical transmission of syphilis, congenital syphilis, testing and diagnosis, public health surveillance, primary health care, general information on syphilis, and other STIs. In this way, the MOOCs present interculturality in the sense of addressing relations to health processes, prevention, and care, respect for differences, diversities, as well as mutual enrichment.

Álvarez-Pérez et al. [22] discussed the development of a pilot project to improve health through digital literacy from electronic sources. The pilot project provided a study with adult and adolescent patients with type 1 and 2 diabetes, in which it was possible to interpret information about the patients’ health online. The approach allowed these people to interact with health professionals, which promoted greater self-management of health conditions, healthier lifestyles, diabetes control, and quality of life. In the context of interculturality, the MOOC included patients from three countries: Spain, Italy, and Sweden, allowing for cultural exchange, using medical systems, improving accessibility, equity, integration of health care, humanization, and continuity of care. It also fostered good practice, interaction, and understanding of the course theme.

Kusnoor et al. [26] described a MOOC carried out by a transdisciplinary working group of clinical researchers, specialists in developing work with groups or communities in situations of vulnerability, educators, and knowledge management scientists. The aim was to improve minority recruitment through effective strategies. Interculturality was contemplated in the MOOC with racial and ethnic minorities who were threatened by the generalization of results in clinical trial recruitment processes.

Patiño-Toro et al. [29] developed a project for deaf and hard-of-hearing individuals. At the time, the MOOCS combined different methodologies and educational resources to cater to this group, using tools and content that facilitated the learning process and social inclusion. In addition, elements of interculturality were used, building values such as respect, citizenship, equality, democracy in education, and human rights.

As for Eglseer [24], the development of the MOOC covered the subject of malnutrition in the elderly, an innovative form of nutritional education to train health professionals to care for this specific group and correct malnutrition immediately and effectively. The course contributed to improving knowledge related to oral food intake, medical nutrition, and multidisciplinary cooperation between health professionals. The contributions were evaluated using pre-tests and post-tests, feedback questionnaires to measure the degree of satisfaction of the participants, performance evaluation, analysis of participation in forums and cooperation activities, interviews and focus groups, and descriptive results. By adopting these multi-faceted evaluation approaches, the researchers had a comprehensive view of the course’s impact and future improvements. As a result of the research, according to the paper, the MOOC improved the malnutrition rate and increased the knowledge of the professionals who deal with that group. In terms of interculturality, it has elements linked to social and cultural factors, as well as good practices, interaction, and understanding of the subject.

Ponnaiah et al. [31] show the development of an introductory MOOC on biomedical research to serve a medical education body in India by presenting knowledge in epidemiology and biostatistics. The intercultural elements and characteristics of the course addressed sustainability issues. In addition, they have enabled inclusive medical education in India capable of catering to racial/ethnic diversity, including low-income participants. According to the authors, with MOOCs already available online for users, it was also possible to share cultural knowledge involving other countries facing epidemiological crises and conditions of social inequality. This has enabled people around the world to access quality education and learning experiences inclusively, without significant geographical or financial barriers.

Sneddon et al. [33], in partnership with the University of Dundee and the British Society for Antimicrobial Chemotherapy (BSAC), developed a MOOC capable of meeting the global need for education to support antimicrobial stewardship in low- and middle-income countries. The course engaged participants with traditional health education methods, supporting clinical practices in an intercultural approach. In this way, the course was accessible to a global multi-professional audience, covering a variety of clinical backgrounds and social interactions, enabling the acquisition of new knowledge and skills for clinical practice. The elements of interculturality related to the study concern social inclusion, cultural diversity, and access to education.

In the study by Gilligan et al. [25], a MOOC was developed for the medical community. The development of the curriculum was influenced by research and experience in line with the European Committee for Standardization (CEN) Workshop Agreement and the main accessibility resources for people with disabilities. The intercultural elements identified involved accessibility for people with disabilities. Issues related to blindness and low vision, deafness and hearing loss, learning difficulties, cognitive limitations, limited movement, speech difficulties, photosensitivity, and combinations of these elements were addressed with the medical community.

Oliveira & Gerhardt [28] developed a MOOC on the worsening of social inequalities in the context of the COVID-19 pandemic, covering the area of public health and the fight against inequalities. The elements of interculturality related to the study, namely social inclusion, cultural diversity, and access to education, allowed for the exchange of information, communication, and knowledge shared with society. They also motivated feelings of collectivity in favor of a more just, equitable, and supportive society, responding to the urgency of dialoguing with society about science and health. Table 5 summarizes the findings based on the literature analyzed in response to RQ02.

In general, the articles in Table 5 presented elements and characteristics of interculturality. As for the characteristics of MOOCs, two points stood out. MOOCs (1) promoted knowledge about the culture of other individuals, emphasizing values such as respect, citizenship, equality, democracy in health education, and human rights, and (2) fulfilled their purpose of being offered to a large audience.

### 3.3. RQ03—Do MOOCs Contribute to Improving Distance Education in Health?

The studies analyzed indicated that MOOCs positively contributed to technology-mediated health education, bringing attractive and up-to-date content to the population and improving the training of health workers. The articles analyzed demonstrated the potential of using MOOCs in the teaching and learning process. In addition, MOOCs integrate digital technologies and innovative content, enhancing this process and enabling interaction between students and teachers in VLEs. The content covered in MOOCs tends to make a positive contribution to the community, providing more knowledge on the proposed topics and with great potential for scalability.

According to the studies analyzed, MOOCs are opportunities of great social value, with the potential to impact distance education in health to improve teaching and encourage institutions to develop MOOCs for different groups through innovative pedagogical practices and learning flexibility [31].

Based on the studies and from an interculturality standpoint, MOOCs have contributed to improving technology-mediated education in the health field, as they have provided dialogs and interrelationships between peoples and cultures, shared knowledge, expertise, and experiences, generating sociocultural recognition.

In the view of Ponnaiah et al. [31], MOOCs contribute to improvements in technology-mediated health education due to the dissemination and multiplication of knowledge, the flexibility of time and space, the supply of variable content, and the stimulation of interaction among participants.

Álvarez-Pérez et al. [22] note that MOOCs advance the dissemination of health information by increasing opportunities to (1) access information when it offers opportunities to access digital health in any geographical location; (2) increase engagement as MOOCs offer interaction between participants, ensuring relevant, understandable, and useful content for the target audience—in this case, healthcare professionals and people with diabetes; (3) offer data analytics and assessment, because this MOOC offers valuable results and information as an educational tool, and (4) provide scalability, due to the scope of the training, reaching a large number of people.

Caitano et al. [11] analyzed MOOCs released during the syphilis epidemic in Brazil, offering insights into the role of distance education in health. The authors observed that extending MOOC reach by leveraging technology could encompass wider audiences compared to traditional educational methods. Their study also demonstrated two aspects related to MOOCs. First, MOOCs promoted access to information on syphilis, prevention, diagnosis, and treatment for individuals across the country, including those in remote areas with limited access to health services. Second, they enhanced community engagement by promoting awareness campaigns and preventive measures on syphilis, social media interaction, and active user participation. Lastly, the authors demonstrated the effectiveness of impact assessments, describing the potential of this approach in reducing disease incidence and improving health outcomes.

In the view of Oliveira & Gerhardt [28], the MOOC contributed to technology-mediated education in health by providing broad access to knowledge about COVID-19 and its implications in terms of public health and inequalities; a focus on health inequities, promoting a deeper understanding of health disparities and encouraging actions to mitigate inequalities; combating infodemic and fake news, promoting public health and preventing the spread of false information; as well as interdisciplinary collaboration, providing an integrated and holistic approach to dealing with public health challenges, enriching the educational experience of course participants.

Kusnoor et al. [26] dealt with the inclusion of minority groups in clinical trials and the promotion of equity in health. The improvements contemplated in this study include broad access, training health professionals, and promoting health equity. The MOOC reported by Patiño-Toro et al. [29] promotes distance education in health for the deaf community, helping to overcome barriers and promoting inclusion and training. In this sense, this MOOC addressed health education components such as accessibility, social inclusion, empowerment, and professional development.

### 3.4. RQ04—What Methodologies Are Employed in MOOC Production, and How Is Interculturality Incorporated into Such Methodologies?

Based on this review, the methodology generally used in the development of MOOCs is action research, which is empirically based social research that is designed and conducted in close association with an intervention or a collective problem resolution. In this process, researchers and participants, representatives of the issue or problem, engage in a cooperative and participatory way [36]. In such a methodology, several strategies have been applied to accomplish the proposed objective, which depends on the MOOC scope and proposal. It was observed that the MOOC production process allows for a dynamic integration between research and practice, enabling course creators to adapt their strategies based on the results obtained along the way. Given this context, Table 6 summarizes the findings based on the literature analyzed in response to RQ04.

The methodologies used in MOOC development have been found to incorporate elements of interculturality in several forms, bringing together professionals from different cultural backgrounds and areas of specialization in a collaborative and interdisciplinary process [13]. This process can include language adaptation, e.g., content translation into several languages, consideration of specific cultural values and practices, and incorporation of examples, feedback, and cases relevant to diverse communities [12].

Such methodologies aimed to promote inclusiveness and accessibility in courses, enabling a wide range of people, irrespective of their cultural background, to benefit from the knowledge shared through MOOCs [33]. Finally, the variety of methodological approaches reflects the wide range of strategies for achieving course objectives based on specific needs. This aspect may also enhance the production of MOOCs and the careful construction of the OERs.

## 4. Discussion

MOOCs are known for their global accessibility, flexible scheduling, variety of content, and innovative teaching approaches, making them a promising tool for educating the public, healthcare providers in training, and practicing healthcare professionals [37]. The production of MOOCs in health is an important strategy for improving medical training in primary health care (PHC), i.e., the first level of health care in Brazil related to low-complexity services. In the context of the Brazilian National Health System (SUS, for its acronym in Portuguese), this strategy can provide interventions related to the reality of the territory [38]. Some examples of this are the interventions implemented by the “More Doctors Program”—a Brazilian government program that seeks to place physicians in regions where there is a shortage or absence of these professionals—and primary health care programmatic actions. Our findings suggest that the use of MOOCs for technology-mediated health training could shape the work of these professionals [39].

From the perspective of Valentim et al. [40], producing MOOCs in the health area involves a series of essential steps to ensure the quality and effectiveness of these courses. In the process, it is essential to carry out pilot tests and continuous evaluations to identify and correct any problems and guarantee the quality of the course. In addition, it is important to consider accessibility, making the course accessible to people with visual, hearing, or motor disabilities.

To summarize the findings of the first of our four RQs, the process of producing MOOCs in the health sector requires careful planning, an appropriate pedagogical approach, and the use of innovative educational technologies to offer quality teaching that is accessible to many people.

In addition, the courses found in the articles analyzed are available on various platforms. The platform of the Virtual Learning Environment of the Brazilian Health System (AVASUS) is an example. As an official platform of the Brazilian Ministry of Health, AVASUS was developed for health professionals and students and aims to improve training, management, and care in the Brazilian National Health System (SUS) [18]. It currently has more than 1,200,000 total registered users, 3,300,000 enrolled users, and more than 420 Active Courses; see https://avasus.ufrn.br/ (accessed on 1 June 2024).

The platform of the Virtual Campus for Public Health (VCPH, available at https://campus.paho.org/) and the Pan-American Health Organization (PAHO/WHO) is also an example of a virtual environment whose digital tools provide access to knowledge and resources essential to public health. The VCPH offers courses in South America, Central America, and the Caribbean in many languages, e.g., Spanish, Portuguese, and English.

Additionally, collaboration among institutions with expertise in technology-mediated education, both national and international, has been a common practice in the production and distribution of MOOCs [22]. This partnership broadens the scope of the courses, enriches their content, and promotes interculturality in a diversity of perspectives, which ensures a more complete and fulfilling educational experience for the participants [41].

Regarding RQ02, MOOCs have applied various elements and characteristics of interculturality to foster dialog and interrelationships between different cultures and groups. This entails the inclusion of content that addresses issues relevant to different communities, respecting their cultural particularities, and promoting the recognition of sociocultural diversity. Alongside this, strategies such as translating into different languages, adapting content, and using culturally relevant examples have been employed to ensure the accessibility and relevance of courses for diverse audiences [28].

Interculturality also plays a key role in the production of MOOCs and the structuring of learning pathways in the healthcare area, as it promotes inclusion and mutual understanding between students from different cultural backgrounds. When developing online courses, it is essential to consider the cultural diversity of the participants, integrating content and pedagogical approaches that are culturally sensitive and relevant. This perspective allows courses to address different health practices and cultural contexts, providing a richer and more inclusive educational experience.

Furthermore, interculturality encourages the exchange of knowledge and experiences between students from different regions, enriching collective learning and fostering a global understanding of health practices. In this way, incorporating interculturality not only improves the quality and effectiveness of MOOCs and learning pathways but also promotes equity and inclusion in the area of health education [27].

Concerning RQ03 and the contribution of MOOCs to continuing health education, the studies analyzed point to a series of benefits. These courses offer attractive, up-to-date, and accessible content that contributes to the ongoing training of health professionals and the dissemination of relevant information to the community [24].

According to Ceccim [42], continuing health education, which includes the use of MOOCs, constantly aims to update practices in line with the latest theoretical, methodological, scientific, and technological knowledge available. Moreover, it entails the construction of relationships and processes that go from within health teams to organizational and inter-institutional/intersectoral practices. This involves not only health professionals but also health institutions and the policies that surround health actions. In the context of public health, continuing health education can have several positive impacts, such as improving the quality of services, preventing and controlling diseases, strengthening the health system, and patient safety, among others. For health students, the impacts are also notable, as their theoretical and practical knowledge is broadened, resulting in a learning process that is more contextualized with the actual demands of health services. This leads to improved technical and behavioral skills, promoting the ability to manage complex situations and make more informed decisions [43].

Over the years, continuing health education has undergone a transformation process of continuous learning regarding professional careers, intending to promote the updating, improvement, and integration of knowledge, skills, and attitudes among health professionals. Approaches to health professional practice require constant adaptation to changes in the fields of medicine, technology, and clinical practice. In short, continuing health education endeavors not only to provide technical knowledge, but also to promote a holistic and collaborative approach to patient care, as well as strengthening commitment to evidence-based practice and professional ethics [44].

Finally, MOOCs can be a powerful tool for public health in various contexts, providing accessible education for health professionals, managers, decision-makers, and the community in general. The reason behind this is due to ongoing education in health by offering relevant content that is varied, flexible, accessible, interactive, adaptable, and applicable, meeting the learning needs of a wide variety of participants in different contexts.

Regarding RQ04 and the methodologies used in the production process of the MOOCs surveyed, a variety of approaches can be observed, but with a general trend towards action research. This methodology allows for a dynamic integration between research and practice, enabling course designers to adjust their strategies based on the results obtained during the process [22]. Various strategies have been employed, such as action research, data and impact analysis, discussions and research, educational studies, evaluation of quantitative and qualitative data, incorporation of elements of interculturality, and interviews [22,24,29].

The production of high-quality MOOCs requires meticulous planning, the integration of innovative educational technologies, an appropriate pedagogical approach, and a pedagogical approach that is adequate for its application. This process is essential to optimize learning in a dynamic online environment, involving text dialogicity, the proposal of reflection activities, and educational resources that capture the student’s attention, such as videos, podcasts, interactives, games, and others.

By providing an inclusive platform for the continuing education of health professionals, MOOCs democratize access to learning and promote the exchange of knowledge and experiences on a global scale. In this way, they contribute to improving the quality of healthcare and training professionals who are better prepared to face the contemporary challenges of the field [24].

## 5. Conclusions

This review found that the production of MOOCs has been conducted comprehensively and collaboratively, thus involving interdisciplinary teams and institutional partnerships to ensure course quality and relevance. In this sense, interculturality is a key element in MOOC development, including content and strategies that respect and promote sociocultural diversity, fostering a more inclusive and enriching educational environment.

Based on this study, MOOCs exert a positive impact on continuing health education. They offer attractive, up-to-date, and accessible content that contributes to the continuing training of health professionals and the dissemination of relevant information to the community. Such courses rely on digital technologies and innovative methodologies to promote social interaction between participants and facilitate access to knowledge in different contexts and locations.

Concerning the limitations reported in the literature, when it comes to the use of MOOCS in the field of health, some strategies should be considered. An example is the inclusion of synchronous activities to increase the depth of interactions between course participants, the adaptation of teaching materials to reflect cultural diversity, and the use of complementary data collection methods, e.g., interviews and focus groups, to obtain a richer and more detailed understanding of intercultural dynamics [1].

In terms of the methodology for content production, MOOCs have adopted a diversified approach, with a general trend towards action research. This methodology allows for a dynamic integration between research and practice, enabling refinements and adjustments throughout course development. Several other strategies were adopted, such as questionnaires, evidence-based research, discussions and surveys, interviews, data and impact analysis, clinical trials, and quantitative and qualitative data evaluation. This was to make the content more compelling and dynamic.

Some limitations should be considered regarding this review’s findings. In particular, the sample of articles analyzed does not represent the full diversity of MOOCs, which restricts the generalization of the conclusions. A further limitation is the reliance on evaluation methods that may not be completely objective. Thus, the focus on interculturality to the detriment of other equally important aspects in MOOC development, combined with uncontrolled variables, may hinder the effectiveness of the analysis. Such limitations are accentuated by the difficulty in generalizing the findings to other contexts or populations. This underscores the need for more comprehensive and methodologically robust future studies that delve deeper into the development and impact of MOOCs in health education.

MOOCs constitute a robust tool for promoting technology-mediated health education. They can potentially train health professionals, disseminate knowledge, and promote inclusion and interculturality. Therefore, continuous collaboration and innovation in MOOC development are essential to ensure their effectiveness and relevance in the contemporary educational scenario. This study could serve as a basis for future trajectories and findings based on further investigations, highlighting the importance of ongoing efforts to improve the quality and impact of MOOCs in health education.

## Figures and Tables

**Table 1:** Research questions.

No.	Description
RQ01	How are MOOCs being developed in the health field, and what are the stages involved in the production process?
RQ02	What are the main elements/characteristics of interculturality applied in MOOC development?
RQ03	Do MOOCs contribute to improving distance education in health?
RQ04	What methodologies are employed in MOOC production, and how is interculturality incorporated into such methodologies?

**Table 2:** Inclusion and exclusion criteria for articles retrieved.

Inclusion Criteria	Exclusion Criteria
IC01—Studies published from 2016 to 2023	EC01—Duplicate articles
IC02—Original articles published in academic journals	EC02—Review articles
IC03—Articles on the area of distance education in health	EC03—Studies not related to distance education in the field of health

**Table 4:** Studies selected for systematic review based on the protocol to search for articles published between 2016 and 2023.

n	Citation	Year	Title	Country
1	Álvarez-Pérez et al. [22]	2022	Co-Creation of Massive Open Online Courses to Improve Digital Health Literacy in Pregnant and Lactating Women	Spain
2	Burlamaqui et al. [23]	2016	Design instrucional para cursos a distância: Um guia para construção de material didático do AVASUS	Brazil
3	Caitano et al. [11]	2022	Massive health education through technological mediation: Analyses and impacts on the syphilis epidemic in Brazil	Brazil
4	Eglseer [24]	2023	Development and evaluation of a Massive Open Online Course (MOOC) for healthcare professionals on malnutrition in older adults	Austria
5	Gilligan et al. [25]	2018	Using MOOCs to Promote Digital Accessibility and Universal Design, the MOOCAP Experience	Ireland, Norway, and Greece
6	Gomes Oliveira et al. [13]	2019	A estruturação de um MOOC para capacitação em pesquisa bibliográfica em bases de dados de enfermagem	Brazil
7	Kusnoor et al. [26]	2021	Design and implementation of a massive open online course on enhancing the recruitment of minorities in clinical trials—Faster Together	United States
8	Longhini et al. [27]	2021	What knowledge is available on massive open online courses in nursing and academic healthcare sciences education? A rapid review	Peru
9	Maxwell et al. [12]	2018	Massive open online courses in U.S. healthcare education: Practical considerations and lessons learned from implementation	United States
10	Oliveira & Gerhardt [28]	2022	O primeiro Curso Aberto, On-line e Massivo (Mooc) sobre COVID-19 e iniquidades no Brasil: potências da saúde coletiva no enfrentamento da infodemia e das fake news	Brazil
11	Patiño-Toro et al. [29]	2023	Proposed methodology for designing and developing MOOCs for the deaf community	Colombia
12	Pessoa et al. [30]	2021	Massive Online Open Course como estratégia para o ensino de segurança no processo de medicação	Brazil
13	Ponnaiah et al. [31]	2022	Design and implementation challenges of massive open online course on research methods for Indian medical postgraduates and teachers -descriptive analysis of inaugural cycle	India
14	Rodrigues & Rossi [32]	2020	Guia de Referência para criar MOOCs: Storytelling integrado ao modelo ADDIE	Brazil
15	Sneddon et al. [33]	2018	Development and impact of a massive open online course (MOOC) for antimicrobial stewardship	Scotland

**Table 5:** Summary of the results to RQ02 based on the literature reviewed.

Author	Course Content	Specific Target Audience	Key Elements of Interculturality				
			SI	CD	CE	AE	AD
Álvarez-Pérez et al. [22]	Development of a pilot project to improve health through digital literacy from electronic sources.	Health professionals	x	x	x	x	
Caitano et al. [11]	Studies on the inclusion of people in vulnerable situations.	LGBT (Lesbian, Gay, Bisexual, Transgender) groups, men who have sex with men (MSM) and people deprived of liberty	x	x		x	
Eglseer [24]	Development of a course on Malnutrition in the elderly.	Nurses, nutritionists, physicians, and other health professionals	x	x		x	
Gilligan et al. [25]	Development of work on digital accessibility and online courses with more in-depth and focused learning topics.	Information and Communication Technology (ICT) professionals	x			x	x
Kusnoor et al. [26]	Development of work to improve the recruitment of minorities (groups or communities in vulnerable situations) through effective strategies.	Health professionals	x	x		x	
Oliveira & Gerhardt [28]	Development of the first MOOC in the field of public health on COVID-19 and health inequities.	Health professionals	x	x		x	
Patiño-Toro et al. [29]	Development of work aimed at adequately assisting people with special needs in educational processes. (Hearing Impairment)	Health professionals	x			x	x
Ponnaiah et al. [31]	Development of a basic course on research methods for postgraduate doctors (PGs) and members of the teaching staff of medical institutions.	Professionals with a medical degree	x	x		x	
Sneddon et al. [33]	Development of a course to meet the global need for education and support antimicrobial stewardship in low- and middle-income countries.	Health education professionals	x		x	x	

Key: SI = Social Inclusion; CD = Cultural Diversity; CE = Cultural Exchange; AE = Access to Education; AD = Accessibility for People with Disabilities; x = indicates the presence of a key element of interculturality in the study.

**Table 6:** Summary of the results to RQ04 based on the literature reviewed.

Authors	Methodological Approach	Methodological Description
Kusnoor et al. [26]	Action Research	Critical analysis of available literature to support the development of courses and underpin their theoretical basis.
Caitano et al. [11], Eglseer [24]	Data and Impact Analysis	Evaluation of the impact of courses on participants’ learning and clinical practice as evidence of their effectiveness.
Maxwell et al. [12], Álvarez-Pérez et al. [22]	Discussions and Research	Collection of feedback and opinions from participants, contributing to the continuous improvement of the courses.
Gilligan et al. [25], Ponnaiah et al. [31], Sneddon et al. [33]	Educational studies	Evaluation of the effectiveness of specific interventions in the courses, ensuring an evidence-based approach.
Gomes Oliveira et al. [13], Pessoa et al. [30]	Evaluation of Quantitative and Qualitative Data	Comprehensive analysis of course results, combining quantitative and qualitative data to achieve deeper insights into the participants’ experience.
Maxwell et al. [12], Gomes Oliveira et al. [13], Sneddon et al. [33]	Incorporation of elements of interculturality	Integration of professionals from different cultural backgrounds and specialties, with adaptation of course content to reflect diverse cultural values and practices.
Maxwell et al. [12]	Interviews	In-depth comprehension of students’ needs and expectations, providing valuable insights for course development.
